# Supplementary material for: Multi-layered dosage compensation of the avian Z chromosome by increased transcriptional burst frequency and elevated translational rates
Source: Nat Commun. 2025 Oct 13;16:9088. doi: 10.1038/s41467-025-64817-w (PMC12518621; doi:10.1038/s41467-025-64817-w)
Supplement: Supplementary file 1 — Supplementary Information [file 41467_2025_64817_MOESM1_ESM.pdf]

Supplementary Information for:

Multi-layered dosage compensation of the avian Z chromosome by increased transcriptional burst frequency and elevated translational rates

Natali Papanicolaou[1,*], Antonio Lentini[1,*], Sebastian Wettersten[1], Michael Hagemann-Jensen[2], Annika Krüger[1], Jilin Zhang[1], Christos Coucoravas[1], Ioannis Petrosian[1], Xian Xin[1], Ilhan Ceyhan[1], Joanna Rorbach[1], Dominic Wright[3], and Björn Reinius[1,#]

[1] Department of Medical Biochemistry and Biophysics, Karolinska Institutet, Stockholm, Sweden.
[2] Department of Cell and Molecular Biology, Karolinska Institutet, Stockholm, Sweden.
[3] AVIAN Behavioural Genomics and Physiology Group, IFM Biology, Linköping University, Linköping, Sweden.
[*] Co-first author, equal contribution.
[#] Correspondence to: bjorn.reinius@ki.se

The following information is included in this file:

Supplementary Figures 1 – 14

Supplementary Figure 1

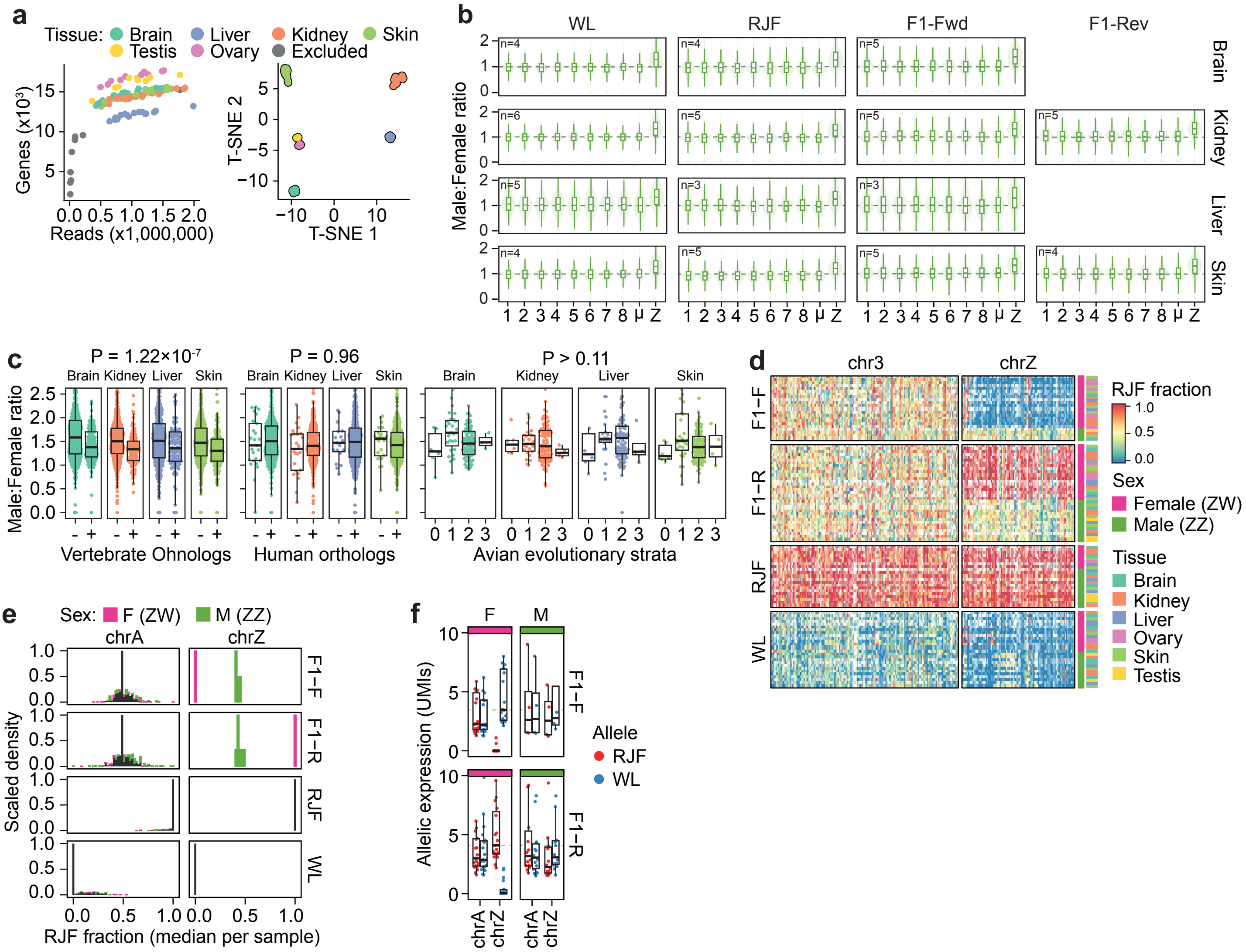

Supplementary Fig. 1. Allele-resolved bulk RNA-seq reveals transcriptional upregulation of the female Z chromosome. **a,** Quality assessment of bulk RNA-seq libraries of tissue samples. On the left: Scatterplot of the number of thousands of genes detected (y-axis) and the number of sequencing reads in millions (x-axis), with each point representing an independent RNA-seq library. Different colours represent libraries prepared from distinct tissue samples (Brain: n=18, Kidney: n = 25, Liver: n=16, Skin: n=22, Ovary: n=12, Testis: n=9) with samples excluded due to low quality represented in grey. On the right: t-distributed stochastic neighbour embedding (t-SNE) of RNA-seq libraries, with each tissue cluster represented in different colours. **b,** Male:Female ratios of gene expression per tissue and cross shown as boxplots over violin plots. Number of samples per tissue (n) shown in respective panels. Only expressed genes (average FPKM >1) were included in the analysis (chr1 =1683-1785, chr2 = 1127-1178, chr3 = 1018-1065, chr4 = 931-977, chr5 = 807-832, chr6 = 434-480, chr7 = 421-450, chr8 = 424-452, chrµ = 5195-5594, chrZ = 596-624). µ denotes grouped micro-chromosomes 9-33. Data shown as median (center line), first and third quartiles (box limits) and 1.5x interquartile range (whiskers). **c,** From left to right: Boxplots of Male:Female gene expression ratios of bulk RNA-seq of chicken tissues for vertebrate ohnologs (671 genes) (+) and non-ohnologs (-), human orthologs (450 genes) (+) and non-orthologs (-) and Z-linked genes belonging to different evolutionary strata (0 = oldest, 3 = newest; 133 genes). Tukey-HSD ANOVA was used for significance testing. Data shown as median (center line), first and third quartiles (box limits) and 1.5x interquartile range (whiskers). **d,** Heatmap of allelic expression for chromosome 3 (389 genes) and chromosome Z (234 genes) for pure (WL = White Leghorn; n=26, RJF = Red Junglefowl; n=21) and forward (F1-F, RJF x WL; n=22) and reverse (F1-R, WL x RJF; n=33) F1-derived tissue samples (total: n = 102). **e,** Histogram of median fraction of allelic red Junglefowl (RJF) reads of bulk RNA-seq of pure (WL; White Leghorn or RJF; Red Junglefowl) or F1 (F: RJF x WL, R: WL x RJF) female and male tissue samples shown as scaled density. **f,** Allelic expression in UMI counts (derived from UMI-containing reads) of bulk UMI-containing RNA-seq (SS2-UMI) for F1 reciprocal cross male and female tissue samples (F1-Forward; RJF x WL; female: n=18, male: n=4; F1-Reverse; WL x RJF; female: n=19, male: n = 14). RJF (Red Junglefowl) and WL (White Leghorn) alleles shown in red and blue respectively. Data shown as median (center line), first and third quartiles (box limits) and 1.5x interquartile range (whiskers).

Supplementary Figure 2

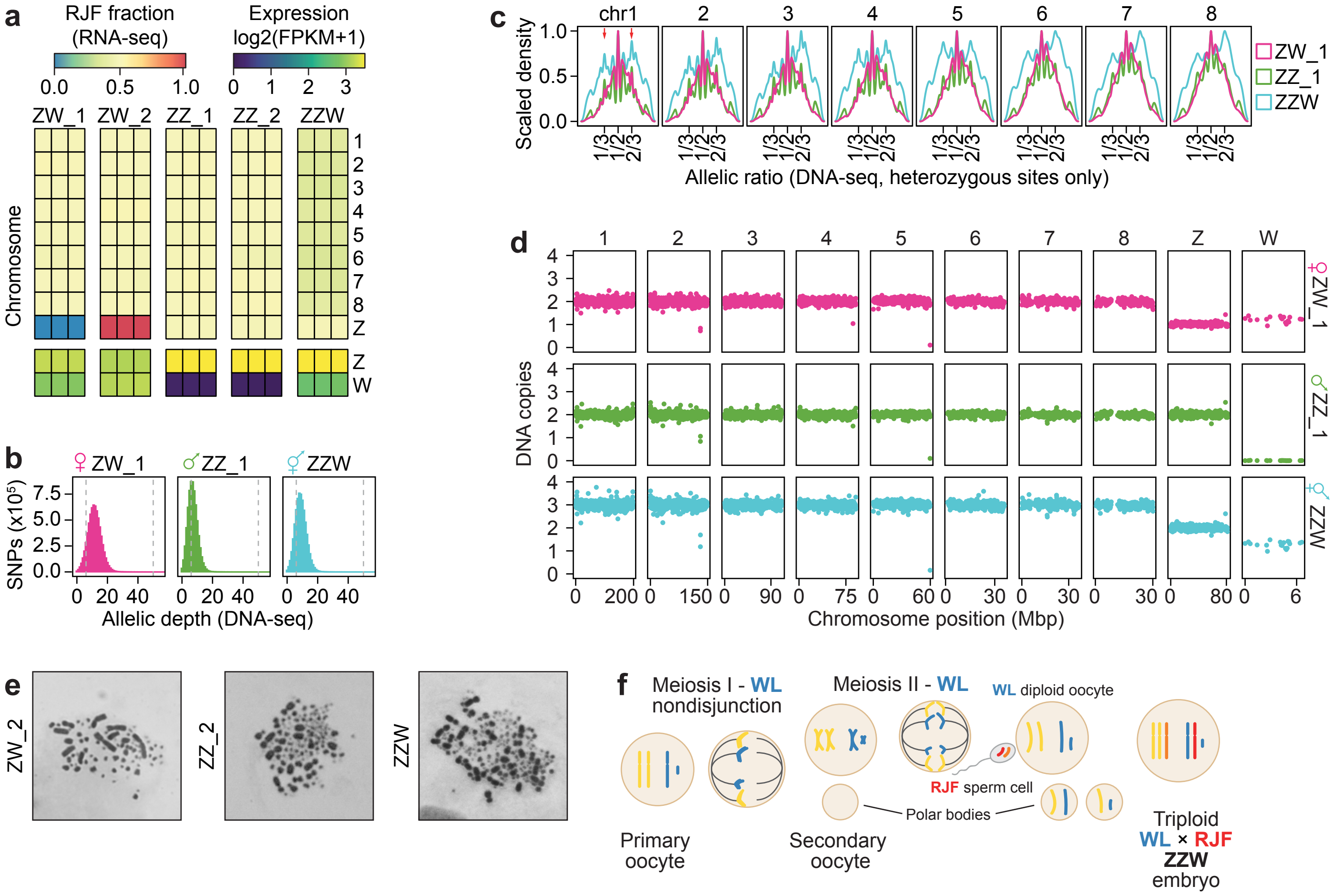

Supplementary Fig. 2. Identification of a triploid intersex (ZZW) sample.
a, Allele expression heatmap for bulk RNA-seq for chromosomes 1-8, Z and W (total of 2943 genes: chr1=629, 2=439, 3=396, 4=376, 5=313, 6=195, 7=173, 8=182, Z=240) shown per sample (F1 primary CEF lines derived from n=2 female, n=2 male and n=1 intersex chicken embryos), coloured by fraction of red Junglefowl (RJF) reads (top) and log2-normalised reads per kilobase million-normalised reads counts for the Z and W chromosomes (bottom). **b,** Histogram plot of number of single-nucleotide polymorphisms (SNPs) detected on the y-axis and allelic coverage of DNA-sequencing libraries on the x-axis. Vertical lines denote cutoffs used. **c,** Scaled density of allelic ratios over heterozygous sites based on DNA-sequencing data for female, male and intersex samples shown in pink, green and teal respectively, for chromosomes 1-8. For diploid F1 samples, allelic ratios are expected at 0.5. Red arrows indicate the unequal distribution of allelic ratios due to the presence of two WL allele copies and one RJF allele copy per chromosome in ZZW intersex samples. **d,** Scatter plots of DNA copy number (y-axis) per chromosomal position in Mbp (x-axis) shown for (macro)chromosomes 1-8, Z and W. Female, male and intersex samples shown in pink, green and teal respectively. **e,** Representative metaphase spreads depicting the karyotypes of a female (ZW_2; n=15 identifiable macrochromosomes), male (ZZ_2; n=16 identifiable macrochromosomes) and intersex (ZZW; n=24 identifiable macrochromosomes) samples from 3 independent experiments. **f,** Schematic representation of the formation of a triploid intersex embryo. During meiosis I, a nondisjunction event occurring at anaphase I in the primary oocyte can lead to the aggregation and transfer of sister chromatids to a single secondary oocyte, generating a void polar body. Meiosis II proceeds to generate two haploid polar bodies and one diploid oocyte which upon fertilisation by a haploid sperm cell generates a triploid zygote. Maternal autosomes colored in yellow, maternal Z and W colored in blue, paternal autosomes colored in orange and paternal Z in red.

Supplementary Figure 3

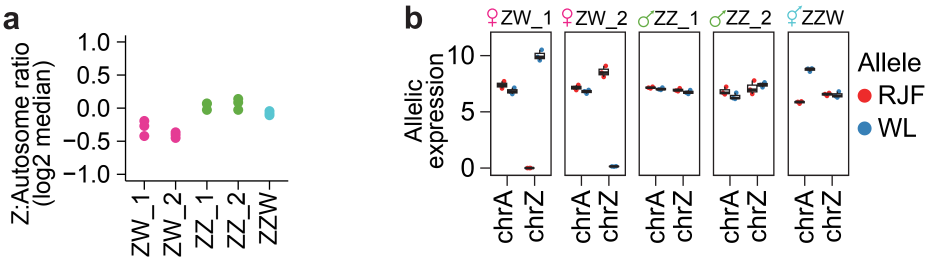

Supplementary Fig. 3. Amplification-free bulk RNA-seq confirms Z-chromosome upregulation of the female Z.
a, Log2-normalised median Z:autosome ratios of gene expression (FPKM>1, total number of genes used in analysis: 10361; autosomal genes = 9888, Z-linked genes=473) per sample (average of 3 technical replicates based F1 primary CEF lines derived from on n=2 female, n=2 male and n=1 triploid intersex chicken embryos) of bulk RNA-seq (Truseq) in CEF lines. Female, male and intersex samples shown in pink, green and teal respectively. **b,** Boxplots of allelic gene expression in FPKM of bulk RNA-seq (Truseq). Average of 3 technical replicates based F1 primary CEF lines derived from on n=2 female, n=2 male and n=1 triploid intersex chicken embryos. Allele-resolved genes included in the analysis = 4956 (average FPKM>1, autosomal genes = 4737, Z-linked genes = 219). RJF and WL alleles shown in red and blue respectively.

Supplementary Figure 4

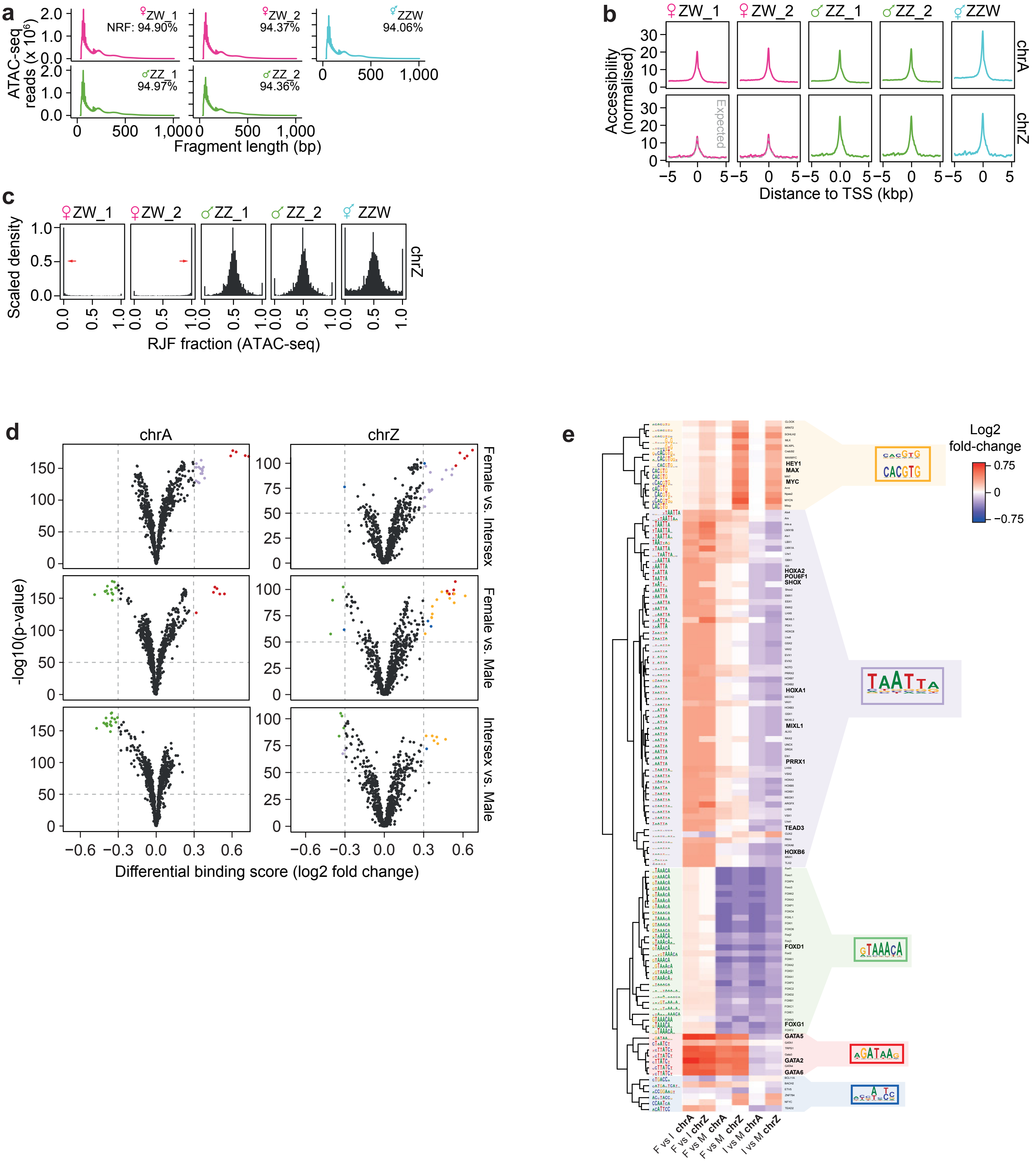

Supplementary Fig. 4. Chromatin accessibility and transcription factor footprinting of the chicken Z chromosome.
a, ATAC-seq library fragment size distribution with fragment length in base pairs on the x-axis and library read numbers on the y-axis, shown as mean of n=4 independent replicates. Female, male and intersex samples shown in pink, green and teal respectively. NRF = non-redundant fraction. **b,** Density plots of normalised accessibility for autosomes (top panel) and Z-chromosome (bottom panel) per primary CEF line (mean of n=4 replicates) with distance from the TSS in kilo base pairs on the x-axis. Female, male and intersex samples shown in pink, green and teal respectively. **c,** Density plots of allelic chromatin accessibility shown as fraction of RJF (red Junglefowl) allelic reads for chromosome Z per primary CEF line. Mean of n=4 independent replicates. Female, male and intersex samples shown in pink, green and teal respectively. Red arrows shown to indicate allelic skew of chrZ in ZW samples. **d,** Volcano plots of the differentially bound transcription factors between sexes for autosomes (chrA) and for chrZ. The x- axis shows the log2 fold change of the differential binding score as calculated by the TOBIAS BINDetect module, and the y-axis represents the significance of the observed change. The most significant transcription factors (>|0.3| change in binding score) for each comparison have been coloured, whereas FOX and GATA transcription factor families, which consistently showed preferential binding on male and female chromosomes respectively, were marked with different colour. Interestingly, a group of transcription factors was detected preferentially bound on both female chrZ and intersex chrZ when compared to the male chrZ, a difference that was not detected in the autosomes. Note that for motifs recognized by more than ten different possible transcription factors, their names are not shown. **e,** Heatmap showing the log2 fold change of the differential binding scores of the transcription factors per comparison (Left to right: Female - Intersex: autosomes, Female - Intersex: ChrZ, Female - Male: Autosomes, Female-Male: ChrZ, Intersex-Male: Autosomes and Intersex-Male: ChrZ). Only transcription factors with differential binding score >|0.3| in at least one comparison were included. The colour indicates the differential binding score, with positive score (red) suggesting preferential binding to the first sex of the comparison (female/intersex) and negative score (blue) indicating preferential binding to the second sex of the comparison (male/intersex). The transcription factors were clustered based on their motif similarity and consensus motifs were created per cluster. The colour-coding corresponds to transcription factors belonging to the same family or recognizing the same motif (shown as seqlogos).

Supplementary Figure 5

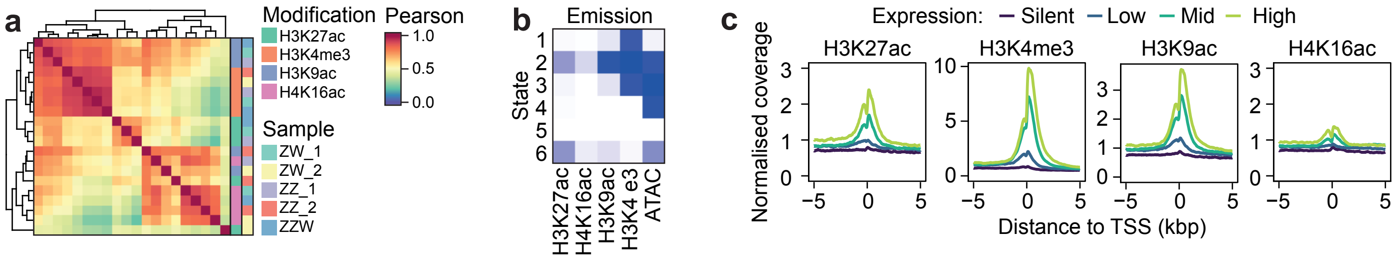

Supplementary Fig. 5. Quantitative ChIP-seq of the chicken Z chromosome.
a, Pearson correlation heatmap of quantitative ChIP-seq histone modifications and samples with hierarchical clustering shown on the right for n=20 samples (5 CEF lines * 4 histone modifications) based on n=71,262 10kb bins. **b,** Heatmap of chromHMM's emission parameters with each row corresponding to a different chromatin state and each column to a different chromatin mark (quantitative ChIP-seq) or chromatin accessibility (ATAC-seq). The colour gradient shows the probability of observing the respective chromatin mark or accessibility in that state, with darker colours corresponding to higher probability and lighter colours to lower probability. **c,** Density plots of normalised coverage of quantitative ChIP of CEFs around genomic transcription start sites (TSS) in kilo base pairs per histone modification, based on 3 technical replicates. The different colours correspond to levels of gene expression (silent, low, mid, high), based on bulk RNA-seq in CEFs.

Supplementary Figure 6

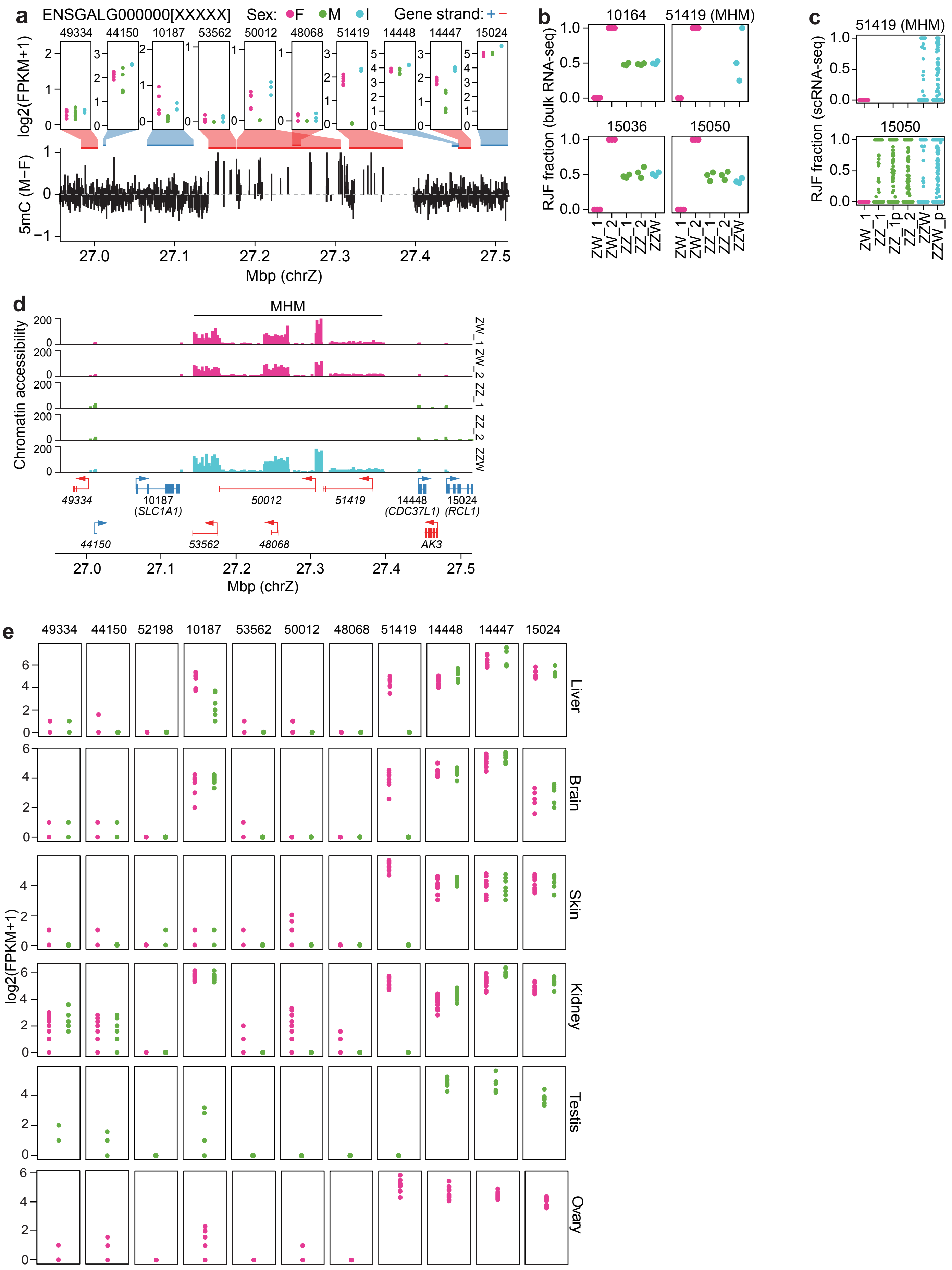

Supplementary Fig. 6. The male hypermethylated (MHM) region is accessible and expressed in female and intersex cells.
a, Top: Log2-normalised gene expression (in fragments per kilobase million, FPKM) of transcripts of the Z-linked male hypermethylated region (MHM) in female, male and intersex samples (derived from n=2 female, n=2 male and n=1 triploid intersex chicken embryos, 3 technical replicates) based on bulk RNA-seq in CEFs. Transcript names shown as "ENSGALG000000[XXXXX]" with the last five digits corresponding to Ensembl transcript IDs appearing above each transcript's panel. Bottom: Male:Female 5mC enrichment in the MHM region. **b,** Allele-resolved expression of transcripts of the MHM region of chrZ displayed as fraction of Red Junglefowl (RJF) reads for each sample based on bulk RNA-seq. Female, male and intersex samples shown in pink, green and teal respectively. **c,** Allele-resolved single-cell RNA-seq shown as fraction of Red Junglefowl (RJF) reads for transcripts of the MHM region for each sample based on scRNA-seq (Smart-seq3) of CEFs (number of cells: ZW_1: n=329, ZZ_1: n=323, ZZ_1p: n=350, ZZ_2: n=366, ZZW: n=364, ZZW_p: n=350, where "_p" denotes CEFs of earlier passage). **d,** Genomic tracks of bulk chromatin accessibility for the Z-linked male hypermethylated (MHM) region [27.1-27.4 Mbp] grouped by sample and coloured by sex, with female, male and intersex samples shown in pink, green, and teal respectively, from ATAC-seq of F1 CEF lines derived from n=2 female, n=2 male and n=1 triploid intersex chicken embryos. Shown as average of n=4 technical replicates. **e,** Log2-normalised gene expression (in fragments per kilobase million, FPKM) of transcripts of the Z-linked male hypermethylated region (MHM) in female and male samples based on bulk RNA-seq in WL, RJF and F1 tissues. Transcript names shown as "ENSGALG000000[XXXXX]" with the last five digits corresponding to Ensembl transcript IDs appearing above each transcript's panel.

Supplementary Figure 7

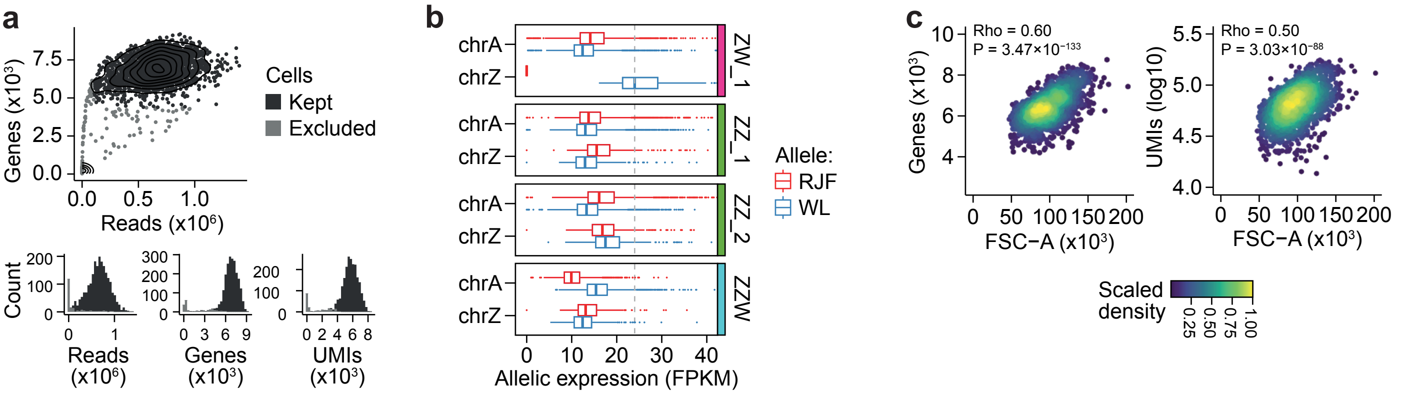

Supplementary Fig.7. Transcriptional burst kinetics of the Z chromosome using Smart-seq3.
a, Quality assessment of single-cell RNA-seq libraries (Smart-seq3). Top: Scatterplot of number of genes detected (in thousands; n=4397-9197 kept genes) on the y-axis in relation to number of sequencing reads (in millions) per sequenced cell. Cells excluded due to low quality shown in grey (kept: n = 2082, excluded: n=200). Bottom: Number of read counts by number of sequencing reads in million (left), detected genes in thousands (middle) and unique-molecular identifiers (UMIs) in thousands (right). **b,** Boxplots of allelic gene expression in FPKM for autosomes (chrA; n=5649 genes) and the Z chromosome (chrZ; n=283 genes) based on single-cell RNA-seq (Smart-seq3) for each CEF line (number of cells per CEF line: ZW_1: n = 329, ZZ_1: n = 323, ZZ_2: n = 366, ZZW: n = 364). The sex of each sample is denoted by a colored bar to the right of each panel (pink=female, green=male, teal=triploid intersex). Red denotes RJF alleles and blue denotes WL alleles. Grey dashed line denotes allelic expression levels of the single female Z chromosome in sample ZW_1. Data shown as median (center line), first and third quartiles (box limits) and 1.5x interquartile range (whiskers). **c,** Left: Scatterplots of number of genes expressed (in thousands) per cell, by cell size (FSC-A) based on FACS and single-cell RNA-seq (Smart-seq3) data. Right: Scatterplot of number of UMIs detected per cell (in log10) in relation to cell size (FSC-A). Colour gradient denotes scaled density. Spearman's Rho and p-value shown over the scatter based on a two-tailed Spearman rank correlation test.

Supplementary Figure 8

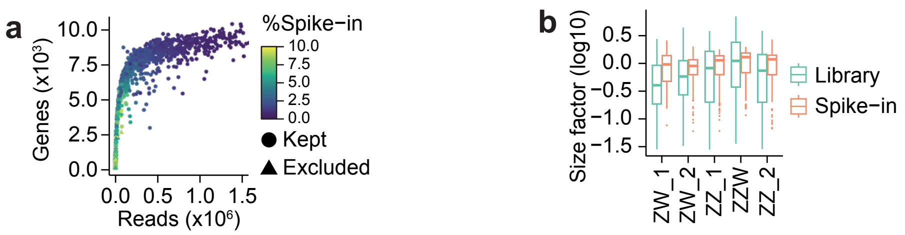

Supplementary Fig.8. Spike-in normalised single-cell RNA-sequencing.
a, Scatterplot of number of genes detected in thousands (y-axis) relative to number of sequencing reads per cell in million (x-axis) based on spike-in single-cell RNA-seq (Xpress-seq). The percentage of spike-ins per cell is denoted by the colour gradient. Cells not passing quality filtering shown as triangles (n kept = 1002, n excluded = 195). **b,** Boxplot of size factor normalisation (in log10) of spike-in single-cell RNA-seq libraries for each CEF line. Data shown as median (center line), first and third quartiles (box limits) and 1.5x interquartile range (whiskers).

Supplementary Figure 9

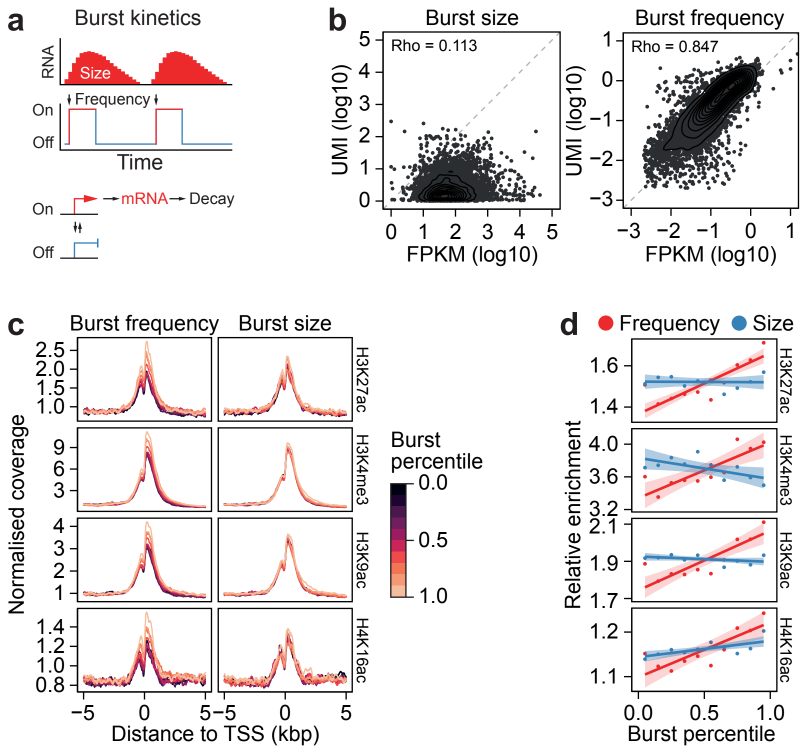

Supplementary Fig. 9. Transcription burst frequency is associated with permissive histone modifications.
a, Schematic representation of transcriptional burst kinetics. During each bursting event, RNA molecules are produced. Top: Transcription burst size represented as number of RNA molecules produced during each transcription burst event (also termed "on" state). Diagram of burst frequency with state on the y-axis ("on" denoting active transcription, "off" denoting no transcription). Bottom: Simplified schematic depicting the transcription burst parameters used to model transcription burst kinetics. **b,** Correlation of UMI-containing reads and FPKM-normalised counts for burst size and burst frequency based on spike-in normalised single-cell RNA-seq (Xpress-seq). Left: Scatter plot showing the correlation between unique molecular identifier (UMI) counts (log10) and fragment per kilobase million (FPKM)-normalised single-cell RNA-seq counts in log10 scale for burst size. Right: Scatter plot showing the correlation between unique molecular identifier (UMI) counts and fragment per kilobase million (FPKM)-normalised single-cell RNA-seq counts in log10 scale for burst frequency. Spearman correlation coefficients (rho) displayed in respective panels. **c,** Quantitative ChIP enrichment around genomic TSS, with distance from TSS in kilo base pairs on the x-axis for each histone modification in CEFs, relative to transcription burst dynamics. Colour gradient denotes the burst percentile, with lighter colours denoting higher degree of burst frequency (left) or size (right), obtained from spike-in single-cell RNA-seq (Xpress-seq) data in CEFs. **d,** Line plots of correlation between quantitative ChIP relative enrichment of each histone modification and transcriptional burst frequency (in red) or burst size (in blue), expressed as burst percentile, obtained from spike-in normalised single-cell RNA-seq (Xpress-seq) in CEFs. Data shown as linear model mean and ± 95% CI.

Supplementary Figure 10

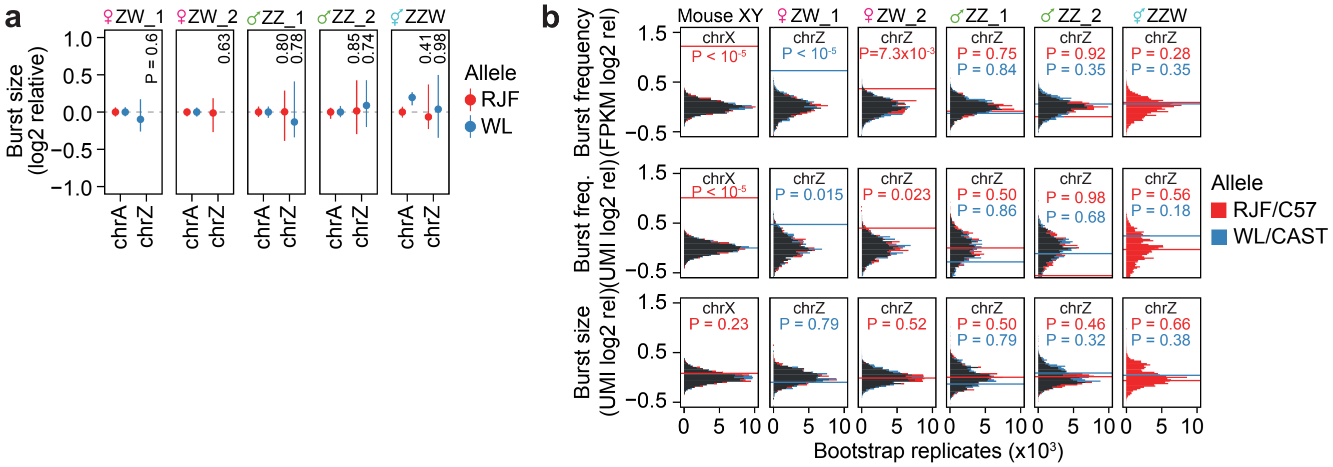

Supplementary Fig. 10. Transcriptional burst kinetics of the chicken Z chromosome mechanistically resemble mammalian X chromosome upregulation.

a, Log2-relative allele-resolved transcription burst size of autosomes (chrA) and the Z-chromosome (chrZ) per CEF line, obtained from spike-in normalised single-cell RNA-seq (Xpress-seq). P-values are empirical P-values from bootstrap resampling with $n=10^5$. Data shown as median ± 95% C.I. **b,** Comparison of transcription burst frequency and size between autosomes and chrX in mouse (chrA genes: n=10025, chrX genes: n=276) or chrZ for chicken (chrA genes: n=7180, chrZ genes: n=332). Histogram of medians of relative burst frequency in FPKM (top) or UMI counts (middle) and burst size in UMI counts (bottom) of randomly subsampled genes, compared to median of chrX for mouse or chrZ for chicken, obtained from single-cell RNA-seq in mouse fibroblasts (n = 682 cells) and CEFs (ZW_1: n=158, ZW_2: n=181, ZZ_1: n=242, ZZ_2: n=242, ZZW: n=179). Number of permutations (bootstrap replicates) shown on x-axis. Red colour denotes C57BL6/J allele in mouse comparisons and RJF in chicken comparisons and blue colour denotes CAST/Eij allele for mouse comparisons and WL for chicken comparisons.

Supplementary Figure 11

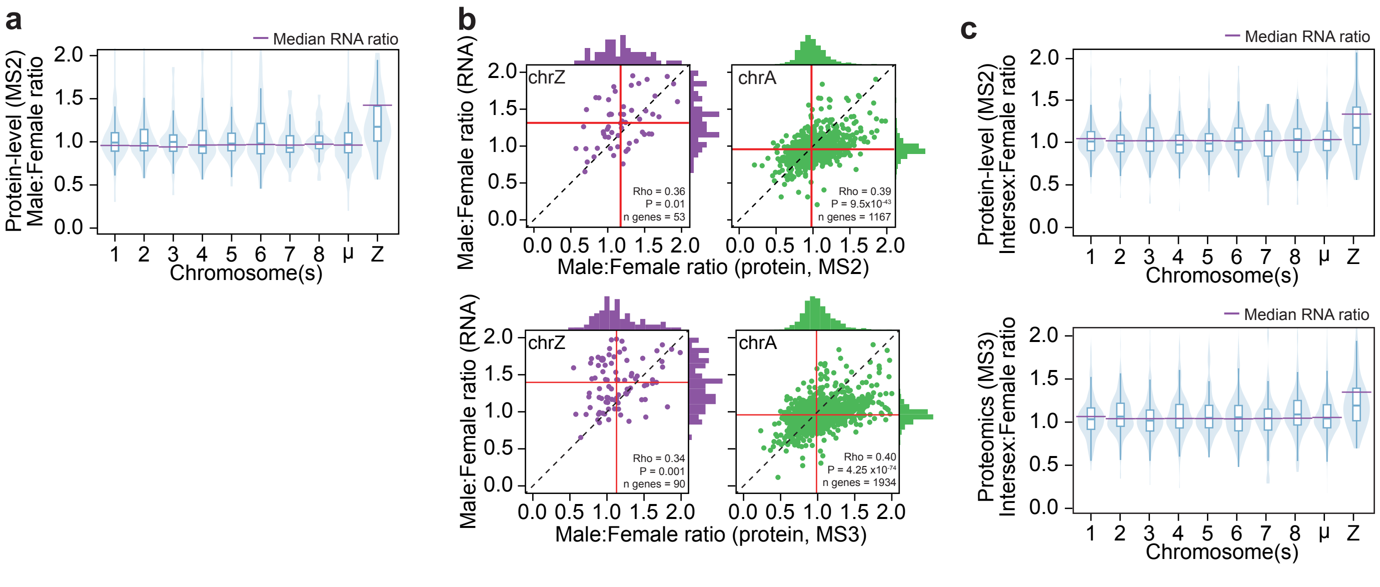

Supplementary Fig. 11. Tandem mass spectrometry reveals significant dosage compensation of the Z chromosome. **a,** Violin and boxplots of Male:Female ratios of protein abundances (MS2: number of proteins per chr: chr1:n =174; chr2: n=109; chr3: n=100; chr4: n=97; chr5: n=100; chr6: n=49; chr7: n=55; chr8: n=49; chrμ: n=621; chrZ: n=58) per chromosome in CEFs, displayed as mean of 3 technical replicates from F1 primary CEF lines derived from n=2 female, n=2 male and n=2 triploid intersex chicken embryos. Purple vertical lines indicate median Male:Female ratios of gene expression based on amplification-free bulk RNA-seq in CEFs. Data shown as median (center line), first and third quartiles (box limits) and 1.5x interquartile range (whiskers). **b,** Top: Scatterplot of Male:Female ratios of protein abundances based on MS2 on the x-axis and amplification-free bulk RNA-seq (Truseq) on the y-axis for Z-linked genes in purple (left) and autosomal genes in green (right), with histograms of distributions shown for MS3 on the x-axis and RNA on the y-axis. Bottom: Scatterplot of Male:Female ratios of protein abundances based on MS3 on the x-axis and amplification-free bulk RNA-seq (Truseq) on the y-axis for Z-linked genes in purple (left) and autosomal genes in green (right), with histograms of distributions shown for MS3 on the x-axis and RNA on the y-axis. Number of genes included in each plot, Rho and p-value based on two-sided Spearman's rank correlation coefficient tests shown at the bottom right corner of each panel. **c,** Violin and boxplots of Intersex:Female ratios of protein abundances based on MS2 (top; number of proteins per chr: chr1:n =174; chr2: n=109; chr3: n=100; chr4: n=97; chr5: n=100; chr6: n=49; chr7: n=55; chr8: n=49; chr9-33: n=621; chrZ: n=58) and MS3 (bottom; number of proteins per chr: chr1:n =264; chr2: n=162; chr3: n=158; chr4: n=134; chr5: n=146; chr6: n=74; chr7: n=79; chr8: n=68; chr9-33: n=945; chrZ: n=91) per chromosome in CEFs, displayed as mean of n=3 independent replicates. Purple vertical lines indicate median Intersex:Female ratios of gene expression based on bulk RNA-seq (Truseq) in CEFs. Data shown as median (center line), first and third quartiles (box limits) and 1.5x interquartile range (whiskers).

Supplementary Figure 12

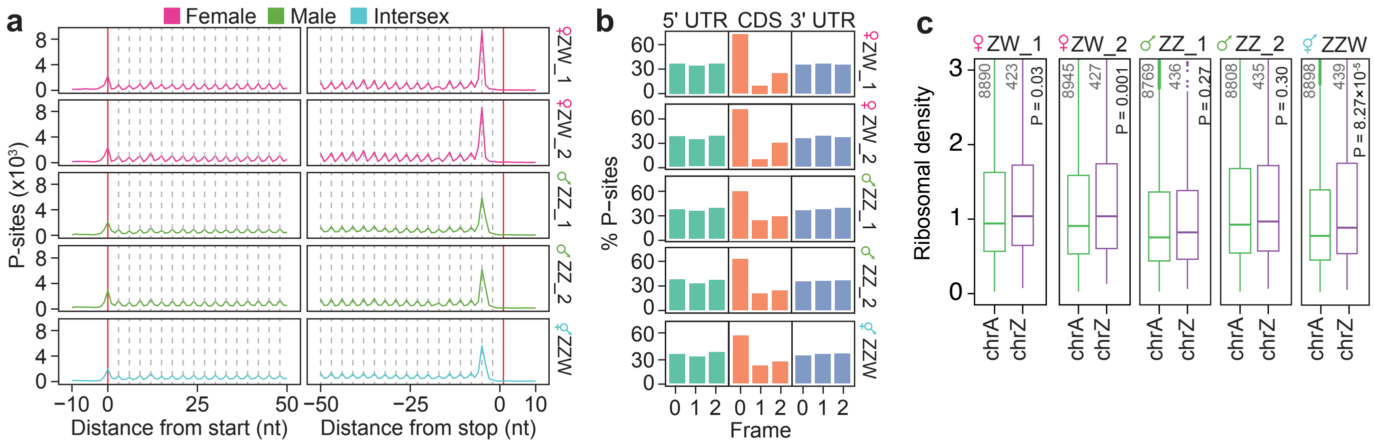

Supplementary Fig. 12. Ribosomal profiling and translational efficiency of Z-chromosome transcripts.
a, Metaprofile plot showing the trinucleotide periodicity along transcript CDS, with distance from start and stop codons in nucleotides on the x-axis and number of P-sites (in thousands) per position on the y-axis for each CEF line. Female samples shown in pink, male samples shown in green and the triploid intersex sample shown in teal. **b,** Bar plot representation of the percentage of ribosomal P-sites per transcript region (5' UTR, CDS and 3' UTR) and codon frame periodicity per CEF line. **c,** Translation efficiency calculated as RPF (ribosome-protected fragment) counts in FPKM normalised by gene expression levels of autosomes (shown in green) and Z-chromosome (shown in purple) per CEF line. The sex of each sample is denoted by the sex symbol at the top part of each panel, in pink for female, green for male and teal for triploid intersex samples. Only genes with RNA FPKM > 1 and RPF FPKM > 1 were included. Two-tailed Mann-Whitney U-test was used for significance testing between the ribosomal densities (translational efficiency) of chrZ vs autosomes. Data shown as median (center line), first and third quartiles (box limits) and 1.5x interquartile range (whiskers).

Supplementary Figure 13

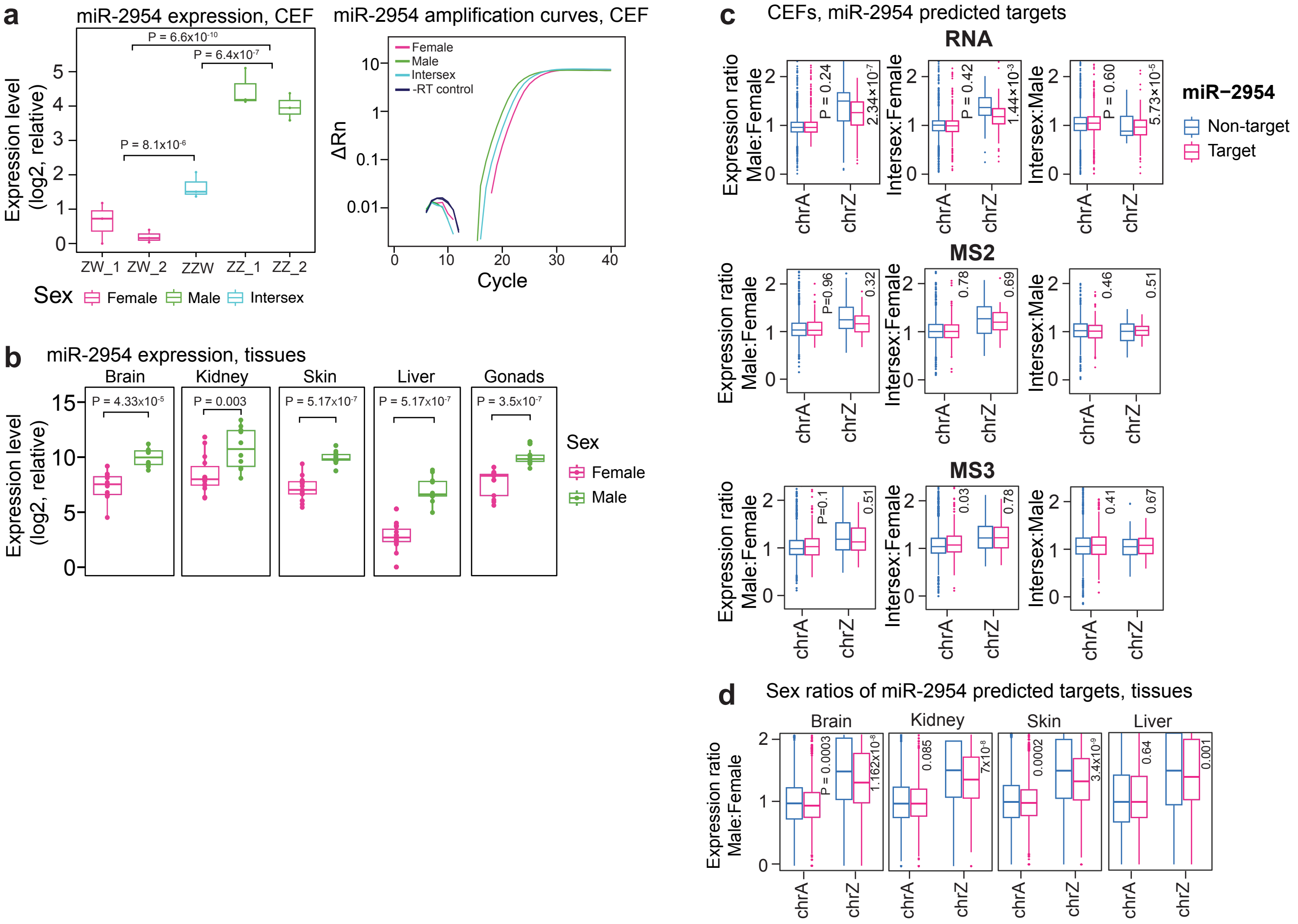

Supplementary Fig. 13. miR-2954-mediated dosage compensation.
a, miR-2954 expression in female, male and intersex fibroblasts quantified by quantitative real time PCR (qRT-PCR). Right: Boxplots of log2-relative expression of miR-2954 in female (ZW_1, ZW_2), male (ZZ_1, ZZ_2) and intersex (ZZW) fibroblasts for n=3 independent experiments shown as relative to sample with the lowest miR-2954 expression. Mann-Whitney-U test used for significance testing (Males-Females, Males-Intersex, Intersex-Females). Left: Amplification curves of female, male and intersex samples, including -RT negative controls in CEFs. Females, males and intersex samples shown in pink, green and teal respectively. Data shown as median (center line), first and third quartiles (box limits) and 1.5x interquartile range (whiskers). Source data are provided as a Source data file. **b,** miR-2954 expression in brain (n = 20, Female: n=10; Male: n=10), kidney (n=24; Female: n=14; Male: n=10), skin (n=25; Female: n=15; Male: n=11), liver (n=25; Female: n=15; Male: n=11) and gonadal tissues (n=23; Female, ovary: n=13; Male, testis: n=11) from pure (White Leghorn; WL or Red Junglefowl; RJF) and reciprocal F1 cross (Forward: RJF x WL; Reverse: WL x RJF) chicken samples. Female and male samples shown in pink and green respectively. Mann-Whitney-U used for significance testing. Source data are provided as a Source data file. **c,** Boxplots of gene expression (RNA; top panel) or protein abundance (MS2, MS3, middle and bottom panels, respectively) ratios for target (RNA: chrA=1147, chrZ=156; MS2: chrA=149, chrZ=27; MS3: chrA=236, chrZ=35) and non-target (RNA: chrA=9303, chrZ=345; MS2: chrA=1028, chrZ=27; MS3: chrA=1709, chrZ=55) genes of miR-2954 with p-values states above autosomal and Z-chromosome boxplots (Mann-Whitney-U test). Expression ratios were calculated for expressed genes (FPKM > 1 or protein abundance > 1). Data shown as median (center line), first and third quartiles (box limits) and 1.5x interquartile range (whiskers). **d,** Boxplots of Male:Female ratios of gene expression (RNA) for target (autosomal targets: n=1344-1407, Z-linked targets: n = 185-191) and non-targets (autosomal non-targets: n=10947-11843, Z-linked non-targets: n = 419-449) of miR-2954 per tissue with p-values states above autosomal and Z-chromosome boxplots (Mann-Whitney-U test). Expression ratios were calculated for expressed genes (FPKM > 1). Data shown as median (center line), first and third quartiles (box limits) and 1.5x interquartile range (whisk

Supplementary Figure 14

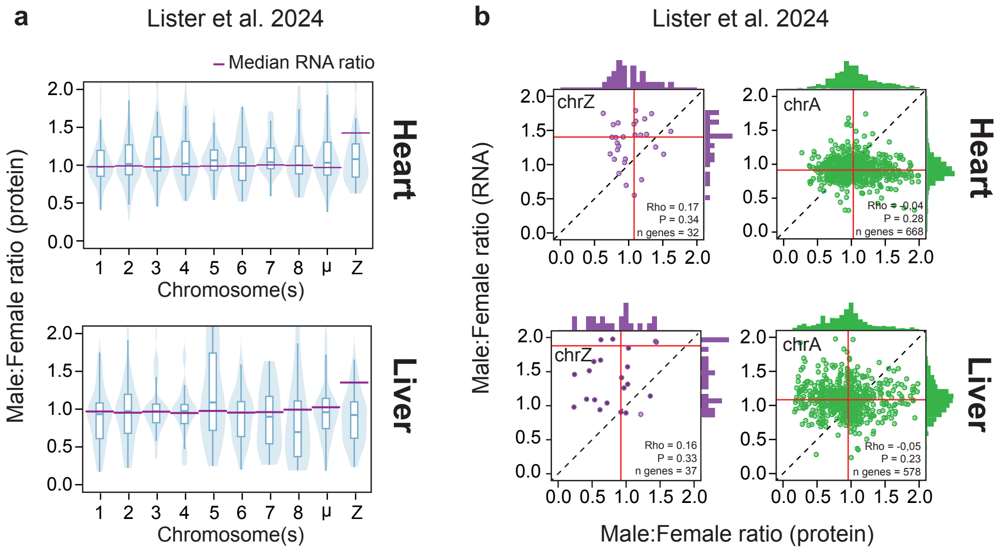

Supplementary Fig. 14. Re-analysis of RNA and proteomic data from Lister et al. 2024.
a, Violin and boxplots of Male:Female ratios of protein abundances based on MS/MS obtained from Lister et al. 2024 for chicken heart (top; number of proteins per chr: chr1: n=103; chr2: n=68; chr3: n=50; chr4: n=57; chr5: n=46; chr6: n = 28, chr7: n=18; chr8: n=20; chr9-33: n=303; chrZ: n=32) and chicken liver (bottom; number of proteins per chr: chr1: n=89; chr2: n=51; chr3: n=45; chr4: n=49; chr5: n=32; chr6: n=27; chr7: n=15; chr8: n=22; chr9-33: n=267; chrZ: n=37) per chromosome, displayed as mean of n=3 independent replicates for chicken heart and n=2 independent replicates for chicken liver. Purple vertical lines indicate median Male:Female ratios of gene expression based on bulk RNA-seq obtained from the respective tissues. Data shown as median (center line), first and third quartiles (box limits) and 1.5x interquartile range (whiskers). **b,** Scatterplots of Male:Female ratios of gene expression based on RNA-seq on y-axis and mass-spectrometry (MS/MS)-based protein abundance measurements on the x-axis for chicken heart (top) and chicken liver (bottom). Only genes detected on both RNA and protein level are included. Red vertical and horizontal lines indicate median protein ratio and median RNA ratio respectively. Number of genes used displayed in respective panels. Number of genes included in analysis, Spearman's Rho and p-value based on two-sided Spearman's rank correlation coefficient tests.
