# Supplementary material for: Multi-layered dosage compensation of the avian Z chromosome by increased transcriptional burst frequency and elevated translational rates
Source: Nat Commun. 2025 Oct 13;16:9088. doi: 10.1038/s41467-025-64817-w (PMC12518621; doi:10.1038/s41467-025-64817-w)
Supplement: Supplementary file 3 — Reporting Summary [file 41467_2025_64817_MOESM3_ESM.pdf]

nature portfolio

Corresponding author(s): BJÖRN REINIUS

Last updated by author(s): Aug 25, 2025

Reporting Summary

Nature Portfolio wishes to improve the reproducibility of the work that we publish. This form provides structure for consistency and transparency in reporting. For further information on Nature Portfolio policies, see our Editorial Policies and the Editorial Policy Checklist.

Statistics

For all statistical analyses, confirm that the following items are present in the figure legend, table legend, main text, or Methods section.

n/a	Confirmed	
☐	☒	The exact sample size (*n*) for each experimental group/condition, given as a discrete number and unit of measurement
☐	☒	A statement on whether measurements were taken from distinct samples or whether the same sample was measured repeatedly
☐	☒	The statistical test(s) used AND whether they are one- or two-sided<br>*Only common tests should be described solely by name; describe more complex techniques in the Methods section.*
☐	☒	A description of all covariates tested
☐	☒	A description of any assumptions or corrections, such as tests of normality and adjustment for multiple comparisons
☐	☒	A full description of the statistical parameters including central tendency (e.g. means) or other basic estimates (e.g. regression coefficient) AND variation (e.g. standard deviation) or associated estimates of uncertainty (e.g. confidence intervals)
☐	☒	For null hypothesis testing, the test statistic (e.g. *F*, *t*, *r*) with confidence intervals, effect sizes, degrees of freedom and *P* value noted<br>*Give P values as exact values whenever suitable.*
☒	☐	For Bayesian analysis, information on the choice of priors and Markov chain Monte Carlo settings
☒	☐	For hierarchical and complex designs, identification of the appropriate level for tests and full reporting of outcomes
☐	☒	Estimates of effect sizes (e.g. Cohen's *d*, Pearson's *r*), indicating how they were calculated

Our web collection on statistics for biologists contains articles on many of the points above.

Software and code

Policy information about availability of computer code

Data collection	Sequencing data was transformed to fastq format using bcl2fastq (v.2.20.0.422) for Illumina sequencing and mgikit (v.0.1.4) for MGI-based sequencing.
Data analysis	Data processing was performed with zUMIs (v.2.9.4c and v.2.9.7 for Xpress-seq data), STAR (v.2.7.2a), bowtie2 (v. 2.5.1), SNPsplit (v.0.4.0), TOBIAS (v0.16.1), Ribowaltz (v.2.0), minute (v.0.6.0), chromHMM (v.1.25), Homer (v5.1), samtools (v1.10), bcftools (v.1.10.2), genmap (v.1.3.0), bedGraphToBigWig (v4), fastp (v.0.20.0), deeptools (v.3.5.4), dnmtools (v.1.4.2), awk (v.5.1.0), HMMcopy (v.1.38.0). Data analysis and plotting was performed in R (v.4.2.2) using the following packages: ggplot2 (v.3.5.2), ggpubr (v0.6.0), data.table (v.1.17.4), ggrastr (v.1.0.2), Biobase (v2.58.0), RColorBrewer (v1.1-3), DESeq2 (v.1.38.3), rtracklayer (v.1.58.0), scran (v.1.26.2), scater (v.1.26.1), ComplexHeatmap (v2.14.0).<br>R custom scripts used for data analysis available at: https://github.com/reiniuslab/Z-upregulation.

For manuscripts utilizing custom algorithms or software that are central to the research but not yet described in published literature, software must be made available to editors and reviewers. We strongly encourage code deposition in a community repository (e.g. GitHub). See the Nature Portfolio guidelines for submitting code & software for further information.

Data

Policy information about availability of data

All manuscripts must include a data availability statement. This statement should provide the following information, where applicable:

- Accession codes, unique identifiers, or web links for publicly available datasets
- A description of any restrictions on data availability
- For clinical datasets or third party data, please ensure that the statement adheres to our policy

Raw and processed sequencing data is available through ArrayExpress and under accessions E-MTAB-14443 (bulk RNA-seq), E-MTAB-14470 (scRNA-seq), E-MTAB-14391 (DNA-seq), E-MTAB-14390 (ATAC-seq), E-MTAB-14392 (ChIP-seq) and E-MTAB-14393 (Ribo-seq). Proteomics data is available through PRIDE under accession PXD054989 (MS2/MS3 proteomics).

Research involving human participants, their data, or biological material

Policy information about studies with human participants or human data. See also policy information about sex, gender (identity/presentation), and sexual orientation and race, ethnicity and racism.

Reporting on sex and gender	Not relevant.
Reporting on race, ethnicity, or other socially relevant groupings	Not relevant.
Population characteristics	Not relevant.
Recruitment	Not relevant.
Ethics oversight	Not relevant.

Note that full information on the approval of the study protocol must also be provided in the manuscript.

Field-specific reporting

Please select the one below that is the best fit for your research. If you are not sure, read the appropriate sections before making your selection.

[x] Life sciences [ ] Behavioural & social sciences [ ] Ecological, evolutionary & environmental sciences

For a reference copy of the document with all sections, see nature.com/documents/nr-reporting-summary-flat.pdf

Life sciences study design

All studies must disclose on these points even when the disclosure is negative.

Sample size	No sample-size calculation was performed before running the experiments.
Data exclusions	RNA-seq libraries failing QC were excluded, otherwise no data exclusion was made.
Replication	All bulk experiments were performed in technical triplicates to ensure reproducibility. All attempts at replication were successful.
Randomization	No randomization of samples were made.
Blinding	Cells were blindly picked by FACS sorting per sex for Smart-seq3 and Xpress-seq experiments.

Reporting for specific materials, systems and methods

We require information from authors about some types of materials, experimental systems and methods used in many studies. Here, indicate whether each material, system or method listed is relevant to your study. If you are not sure if a list item applies to your research, read the appropriate section before selecting a response.

Materials & experimental systems

n/a	Involved in the study
☐	☒ Antibodies
☐	☒ Eukaryotic cell lines
☒	☐ Palaeontology and archaeology
☐	☒ Animals and other organisms
☒	☐ Clinical data
☒	☐ Dual use research of concern
☒	☐ Plants

Methods

n/a	Involved in the study
☐	☒ ChIP-seq
☐	☒ Flow cytometry
☒	☐ MRI-based neuroimaging

Antibodies

Antibodies used: H3K4me3 [Millipore; 04-745], H3K27ac [Active Motif; 39034], H3K9ac [Active Motif; 39137-AF], H4K16ac [Millipore; 07-329]

Validation: H3K4me3 [Miilipore; 04-745]: Validated by the manufacturer through Western Blot and immunohistochemistry and recommended by the manufacturer of the MINUTE-ChIP kit used in this study (Epigenica).
H3K27ac [Active Motif; 39034]: We did not perform cross-validation of this antibody's reactivity. However, it is predicted to have a wide range of reactivity by the manufacturer and its specificity has been validated through ChIP-seq, ChIP-qPCR, immunofluorescence, Western blot and dot plot analyses by the manufacturer.
H3K9ac [Active Motif; 39137-AF]: We did not perform cross-validation of this antibody's reactivity. However, it is predicted to have a wide range of reactivity by the manufacturer and its specificity has been validated through dot blot analysis, peptide array analysis and ChIP analysis.
H4K16ac [Millipore; 07-329]: Validated by the manufacturer through Dot blot analysis, ChIP and immunocytochemistry. Recommended use in our study by the manufacturer of the MINUTE-ChIP kit utilized in our study (Epigenica).

Eukaryotic cell lines

Policy information about cell lines and Sex and Gender in Research

Cell line source(s): We used primary Chicken Embryonic Fibroblast (CEF) cell lines which we derived from 10-13-day old chicken embryos as described in the Methods. No commercial cell lines were used in the study.

Authentication: gDNA sequencing, RNA sequencing.

Mycoplasma contamination: The cell lines were not tested for mycoplasma contamination.

Commonly misidentified lines (See ICLAC register): None.

Animals and other research organisms

Policy information about studies involving animals; ARRIVE guidelines recommended for reporting animal research, and Sex and Gender in Research

Laboratory animals: Red Junglefowl chicken (Gallus gallus, n=21), White Leghorn (Gallus gallus domesticus, n= 26), F1 reciprocal crosses: F1-forward (WL x RJF, n=33), F1-reverse (RJF x WL, n=22).

Wild animals: No wild animals were used in this study.

Reporting on sex: Both sexes were considered in the study design. For adult chickens used in the study, sex was determined based on phenotype. For the primary chicken embryonic fibroblast lines generated from F1 generation chicken embryos, the sex was confirmed using DNA-sequencing. We used differential gene expression analysis to determine whether the Male:Female ratios used in this study as a means of studying Z-chromosome dosage compensation were influenced by sex-specific expression, which we have shown not to be the case (FDR > 0.05, GSEA Biological processes). Number of female and male samples as follows: WL: 14 females, 12 males. RJF: 8 females, 13 males. F1-Forward (WL xRJF): 14 males, 19 females. F1-Reverse (RJF x WL): 4 males, 18 females.

Field-collected samples: None

Ethics oversight: All animal experimental procedures were performed in accordance with Karolinska Institutet’s and Linköpings Universitet’s guidelines and approved by the Swedish Board of Agriculture (permit 16110-2020 Jordbruksverket).

Note that full information on the approval of the study protocol must also be provided in the manuscript.

Plants

Seed stocks | None

Novel plant genotypes | None

Authentication | None

ChIP-seq

Data deposition

☒ Confirm that both raw and final processed data have been deposited in a public database such as GEO.

☒ Confirm that you have deposited or provided access to graph files (e.g. BED files) for the called peaks.

Data access links
May remain private before publication.

BED files: deposited in Zenodo: https://doi.org/10.5281/zenodo.16943266
Total peak count matrices: https://www.ebi.ac.uk/biostudies/preview/ArrayExpress/studies/E-MTAB-14392?query=E-MTAB-14392%20
Raw data (fastq): https://www.ebi.ac.uk/ena/browser/view/ERP163588

Files in database submission

Accession number: E-MTAB-14392 includes the following files:
total_peak_H3K9ac_counts.tsv.gz, total_peak_H3K27ac_counts.tsv.gz, total_peak_H3K4me3_counts.tsv.gz, total_peak_H4K16ac_counts.tsv.gz, total_genebody_enrichment.mat.gz, sample_annotations.txt

Genome browser session (e.g. UCSC)

No genome browser session was used for analysis.

Methodology

Replicates

Three replicates were included for each histone modification examined, with high degree of agreement between them as shown in Supplementary Figure 5a.

Sequencing depth

MINUTE-ChIP library sequencing was performed on a MGI G400RS instrument with paired-end settings (PE100, 100bp per read).

Antibodies

The following antibodies were used in this study:
H3K4me3 [Millipore; 04-745; clone MC315, monoclonal], H3K27ac [Active Motif; 39034; polyclonal], H3K9ac [Active Motif; 39137-AF, rabbit polyclonal], H4K16ac [Millipore; 07-329; rabbit polyclonal].

Peak calling parameters

macs3 callpeak -g 1058535536 --broad

Data quality

No differential peak analysis was performed.

Software

Quantitative ChIP-seq was analysed using the Minute pipeline (https://github.com/elsasserlab/minute) and downstream analysis was performed using deepTools (https://deeptools.readthedocs.io/en/latest/). Computational code is available in Github: https://github.com/reiniuslab/Z-upregulation

Flow Cytometry

Plots

Confirm that:

☐ The axis labels state the marker and fluorochrome used (e.g. CD4-FITC).

☐ The axis scales are clearly visible. Include numbers along axes only for bottom left plot of group (a 'group' is an analysis of identical markers).

☐ All plots are contour plots with outliers or pseudocolor plots.

☐ A numerical value for number of cells or percentage (with statistics) is provided.

Methodology

Sample preparation

F1 primary chicken embryonic fibroblasts were grown to 80% confluency before being FACS-sorted into 384-well lysis plates for Smartseq3 and Xpress-seq. Upon reaching 80% confluency, the media was removed and the cells were washed twice with 1x PBS. The cells were dissociated using 2ml of TryPLE dissociation reagent per 10cm cell culture dish. Once most cells had detached, 2 ml of pre-warmed complete media was added and the samples were centrifuged at 300 x g for 5 minutes to

	pellet the cells. The cell pellets were washed twice in 2 ml 1x PBS to remove all remaining media and TryPLE. Before sorting, the cell pellets were diluted in 5 ml 1x PBS and strained through a 30um strainer to ensure no clumps were present in the cell solution.
Instrument	For Smart-seq3, cells were single-cell sorted using a FACS Aria II instrument. For Xpress-seq, cells were single-cell sorted using a Sony SH800S instrument.
Software	Software used to collect Smart-seq3 FACS data: BD FACSDiva<br>Software used to collect Xpress-seq FACS data: SH800 Software<br>No further analysis was performed other than selection of single-cells during sorting.
Cell population abundance	No cell population abundance analysis was performed as FACS was solely used to collect and sort single-cells into well-plates for scRNA-sequencing.
Gating strategy	*Describe the gating strategy used for all relevant experiments, specifying the preliminary FSC/SSC gates of the starting cell population, indicating where boundaries between "positive" and "negative" staining cell populations are defined.*

☐ Tick this box to confirm that a figure exemplifying the gating strategy is provided in the Supplementary Information.
