# Supplementary material for: Multi-layered dosage compensation of the avian Z chromosome by increased transcriptional burst frequency and elevated translational rates
Source: Nat Commun. 2025 Oct 13;16:9088. doi: 10.1038/s41467-025-64817-w (PMC12518621; doi:10.1038/s41467-025-64817-w)
Supplement: Supplementary file 4 — Description of Additional Supplementary Files [file 41467_2025_64817_MOESM4_ESM.pdf]

Description of Additional Supplementary Files

Supplementary Data 1

Description: Table of differential expression between female and male samples corrected for tissue type using DEseq2.

Supplementary Data 2. Table of differential Zlinked gene expression in pure RJF and WL breeds, corrected for tissue type, using DESeq2 LRT test.

Supplementary Data 3

Description: Table of differential expression presented as pairwise comparisons between male, female and intersex chicken embryonic fibroblasts using DESeq2 LRT test.

Supplementary Data 4

Description: Table of differential transcription factor footprinting using TOBIAS based on ATAC-seq data presented as pairwise comparisons between male, female and intersex chicken embryonic fibroblasts for autosomes and the Z chromosome.

Supplementary Data 5

Description: Results E-box motif enrichment analysis using HOMER, including length in non-N-masked bases per chromosome, number of motifs found per chromosome (E-box number) and ratio of Ebox motif per non-N-masked base.
